# Intrauterine Infusion and Hysteroscopic Injection of Autologous Platelet-Rich Plasma for Patients with a Persistent Thin Endometrium: A Prospective Case–Control Study

**DOI:** 10.3390/jcm13102838

**Published:** 2024-05-11

**Authors:** Tzu-Ning Yu, Tsung-Hsien Lee, Maw-Sheng Lee, Yi-Chun Chen, Chung-I Chen, En-Hui Cheng, Pin-Yao Lin, Chun-Chia Huang, Chun-I Lee

**Affiliations:** 1 Institute of Medicine, Chung Shan Medical University, Taichung 402, Taiwan; ningsyu@gmail.com (T.-N.Y.); jackth.lee@gmail.com (T.-H.L.); msleephd@gmail.com (M.-S.L.); 2 Division of Infertility, Lee Women’s Hospital, Taichung 406, Taiwan; alice4918430@gmail.com (Y.-C.C.); cci1959@me.com (C.-I.C.); enhuicheng@gmail.com (E.-H.C.); jellylin0607@gmail.com (P.-Y.L.); agarhuang@gmail.com (C.-C.H.); 3 Department of Obstetrics and Gynecology, Chung Shan Medical University Hospital, Taichung 402, Taiwan

**Keywords:** autologous platelet-rich plasma, intrauterine infusion, hysteroscopic injection, thin endometrium, frozen-thaw embryo transfer

## Abstract

**Objectives**: To evaluate the effect of intrauterine infusion and hysteroscopic injection of autologous platelet-rich plasma (PRP) in patients with a persistent thin endometrium (EM) undergoing euploid frozen embryo transfer (EFET) cycles. **Methods**: This prospective case–control study enrolled 116 infertile women with thin EM (<7 mm) who underwent hormone replacement therapy (HRT) for EFET. These women had experienced at least one previous unsuccessful EFET cycle, which either resulted in the cancellation of the cycle or failure of pregnancy. A total of 55 women received an intrauterine infusion of PRP before FET, 38 received a hysteroscopic injection of PRP, and 23 received standard HRT treatment without PRP (control group). Only euploid embryos were transferred in these cycles. The primary outcomes were the implantation rate (IR) and clinical pregnancy rate (CPR) after EFET. **Results**: After receiving intrauterine infusion and hysteroscopic injection of PRP, 78.2% and 55.3% of patients, respectively, showed an EM thickness exceeding 7 mm, followed by embryo transfer. The hysteroscopic injection group demonstrated significantly higher IR (52%), a higher trend of CPR (52%), and a higher live birth rate (38%) than the control group (18%, 22%, and 4%). **Conclusions**: Intrauterine infusion and hysteroscopic injection of autologous PRP may be effective methods to increase EM thickness in HRT cycles. According to our results, both methods could increase EM thickness, while hysteroscopic injection appeared to provide more significant assistance in increasing IR, CPR, and live birth rate after EFET in patients with persistent thin EM.

## 1. Introduction

The endometrial (EM) thickness is one of the predictor factors of the success of assisted reproductive technology (ART), including the pregnancy rate, spontaneous abortion rate, and live birth rate [1]. When the EM thickness was less than 7 mm, there was a significant decrease in the implantation rate (IR), clinical pregnancy rate (CPR), and live birth rate [2,3,4]. Therefore, an EM thickness of 7 mm is generally considered a cut-off value for embryo transfer, including in fresh IVF cycles and frozen embryo transfer (FET) [5,6]. FET cycles are usually canceled if the EM thickness remains persistently thin despite prolonged hormone therapy. Many adjuvant therapies are administered to patients with a thin EM, but there is still insufficient evidence to support the use of any specific adjuvant therapy [7].

Autologous platelet-rich plasma (PRP) is prepared from the patient’s whole blood, resulting in a 4–5-fold increase in the platelet concentration [8]. It contains growth factors in the platelet α-granules, including platelet-derived growth factor (PDGF), endothelial growth factor (EGF), transforming growth factor (TGF), vascular endothelial growth factor (VEGF), insulin-like growth factor (IGF), connective tissue growth factor (CTGF), hepatocyte growth factor (HGF), and both anti-inflammatory and proinflammatory cytokines such as interleukin (IL)-4, IL-8, IL-13, IL-17, tumor necrosis factor (TNF)-α, interferon (IFN)-α and stromal cell-derived factor 1-α [9,10,11,12,13]. These factors enable PRP to stimulate cell migration, proliferation, differentiation, and angiogenesis, leading to tissue regeneration [10]. Autologous PRP is widely used in various medical conditions, including orthopedics, ophthalmology, surgery, and wound healing [14]. In the field of obstetrics and gynecology, PRP can improve female sexual dysfunction and promote tissue health in the vagina, bladder, and pelvic floor [15]. In addition, PRP has also been gradually applied in the field of ART, such as intraovarian injection of PRP to improve ovarian function, increase the number of retrieved mature oocytes, enhance embryo quality, and increase the pregnancy rate after embryo transfer in patients with repeated implantation failure [15,16,17,18,19,20].

In an in vitro study, active PRP stimulated the migration of human endometrial epithelial cells, stromal fibroblasts, and mesenchymal stem cells, and additionally, activated PRP increased the proliferation of endometrial stromal cells and mesenchymal cells [21]. PRP increased the EM thickness and improved the EM receptivity through the effect of cytokine regeneration [22]. The first report on the intrauterine infusion of autologous PRP for patients with persistent thin EM was published in 2015, demonstrating the efficacy of PRP treatment in increasing EM thickness and improving pregnancy rates after FET [23]. Subsequently, several studies have been conducted, involving an increasing number of patients who received at least one intrauterine infusion of PRP during the same cycle. Intrauterine infusion of PRP is a relatively easy, quick, and non-invasive method for treating thin EM. These studies have consistently shown the effectiveness of intrauterine infusion of autologous PRP in FET cycles and even in intrauterine insemination cycles [14,24,25,26,27,28].

Hysteroscopic injection of autologous PRP was first described in 2020 [12]. It was hypothesized that when PRP was injected directly into the subendometrial region, the cytokines and growth factors presented in PRP would stimulate the growth of stem cells and promote angiogenesis and cell growth directly. However, further studies are still needed to investigate the underlying mechanism of PRP treatment.

In this study, we aimed to analyze the efficacy of autologous PRP treatment in patients with thin EM during their FET cycles. Specifically, we compared the effectiveness of intrauterine infusion and hysteroscopic injection of PRP. Only euploid embryos were selected for transfer in this study to eliminate the influence of embryo aneuploidy. We assessed the EM thickness before and after PRP treatment and analyzed the associated pregnancy outcomes. The primary objective of this study was to evaluate the implantation rate (IR) and clinical pregnancy rate (CPR) after PRP treatment for patients with persistent thin EM.

## 2. Materials and Methods

### 2.1. Patient Selection

This prospective case–control study involved infertile women undergoing controlled ovarian hyperstimulation (COH) for the preimplantation genetic testing for aneuploidy (PGT-A). The study focused on women with thin EM (<7 mm) who were undergoing hormone replacement therapy (HRT) for euploid frozen embryo transfer (EFET) at Lee Women’s Hospital in Taichung, Taiwan. Recruitment took place between March 2018 and October 2022. All participants had previously experienced at least one unsuccessful EFET cycle, which included either cancellation of the EFET cycle due to thin EM or failure to achieve pregnancy after receiving euploid embryo transfer. All patients included in our study underwent hysteroscopic examination prior to entering the HRT EFET cycle to rule out uterine pathologies, including intrauterine adhesions and Asherman syndrome. Exclusion criteria comprised stage III or IV endometriosis, ovarian failure, hematologic disorders with a platelet count less than 150,000/μL, or anemia with hemoglobin levels below 11 g/dL. As preparing PRP required drawing blood of at least 20 mL, in order to avoid discomfort or concerns among patients, we excluded patients with anemia. We also included a regular group consisting of 30 patients who received standard HRT for EFET and had EM thickness ≥ 7 mm. The indications for the PGT-A were similar to those stated in our previous report, including advanced maternal age, recurrent miscarriage, and repeated implantation failure [29]. The primary outcomes were the implantation rate (IR) and clinical pregnancy rate (CPR) after EFET. The study protocol was approved by the Institutional Review Board of Chung Shan Medical University Hospital (CS-18028) and written informed consent to participate in this study was obtained from each participant.

### 2.2. Controlled Ovarian Stimulation (COH) Protocol

The COH protocol utilized in this study involved a standard GnRH agonist long protocol using leuprolide acetate (Lupron^®^; Takeda Chemical Industries, Tokyo, Japan), a standard GnRH antagonist protocol using cetrorelix acetate (Cetrotide^®^; Merck-Serono, Darmstadt, Germany), or progestin-primed ovarian stimulation (PPOS) with medroxyprogesterone acetate (Provera^®^; Pfizer, Milan, Italy). The choice of protocol was based on the physicians’ preference and aligned with our previous report [29]. Follitropin alfa (Gonal-f^®^; Merck-Sereno, Darmstadt, Germany) and/or hMG (Menopur^®^; Ferring, Pymble, NSW, Australia) were administered for COH starting from cycle day 3 until more than two dominant follicles reached a threshold diameter (≥17 mm). To trigger final oocyte maturation, recombinant human chorionic gonadotropin (hCG) (Ovidrel^®^; Merck-Sereno, Darmstadt, Germany) and/or triptorelin (Decapeptyl^®^; Ferring, Kiel, Germany) were injected. Ovum pick-up was performed approximately 36 h after the trigger injection.

### 2.3. Embryo Biopsy for Preimplantation Genetic Testing for Aneuploidy (PGT-A)

The procedures for oocyte pick-up, in vitro fertilization, embryo culture, biopsy, vitrification, and thawing were conducted following a similar protocol as outlined in our previous report [29]. Briefly, oocytes and embryos were cultured in a tri-gas incubator (5% O_2_, 5% CO_2_, and 90% N_2_) at a temperature of 37 °C. After fertilization through conventional insemination or intracytoplasmic sperm injection (ICSI), individual embryos were transferred into a culture dish containing equilibrated cleavage medium (Quinn’s Advantage^TM^, SAGE Biopharma^®^, Cambridge, MA, USA) within the tri-gas incubator. At 70–72 h after fertilization, the culture medium was changed to an equilibrated blastocyst medium (Quinn’s Advantage^TM^, SAGE Biopharma^®^, USA). Only expanded blastocysts, graded as AA, AB, AC, BA, BB, BC, CA, or CB using the Gardner grading system [30], were selected for trophectoderm biopsy. Trophectoderm cells were gently aspirated, comprising five to ten cells detached from the zona pellucida. The biopsied trophectoderm cells were transferred to new droplets of PBS and rinsed multiple times before being transferred into an RNAse–DNAse-free PCR tube. The biopsied blastocysts were returned to the tri-gas incubator for at least 3 h. The Cryotech vitrification method (Cryotech^®^, Tokyo, Japan) was employed for blastocyst vitrification.

### 2.4. Examination of Diploid–Aneuploid Levels by Next Generation Sequencing

The PGT-A protocol employed in this study was similar to that described in our previous report [29]. Genomic DNA was extracted and amplified using the SurePlex DNA Amplification System (Illumina, San Diego, CA, USA). The amplified DNA product was utilized to generate genomic DNA libraries following the VeriSeq PGS workflow (Illumina, USA). Data analysis was performed using the BlueFuse Multi Software (Illumina, USA), and at least two technicians assessed the diploid–aneuploid levels of each sample. Blastocysts were categorized into three groups based on diploid–aneuploid mosaic ratios determined using the high-resolution next generation sequencing (hr-NGS) platform on biopsied cells [31,32,33]: (i) euploid blastocysts with mosaicism levels ≤ 20%; (ii) mosaic blastocysts with mosaicism levels between >20% and <80%; and (iii) aneuploid blastocysts with mosaicism levels ≥ 80%.

### 2.5. Endometrial Preparation and Euploid Frozen-Thaw Embryo Transfer (EFET)

The endometrium was prepared by administering programmed hormone replacement therapy (HRT) for frozen-thaw embryo transfer, as previously described [34]. Patients began daily oral estradiol valerate (Estrade^®^, Synmosa, Taipei, Taiwan) from the menstrual cycle day 3. On cycle days 11 to 13, transvaginal ultrasound was used to measure the endometrial (EM) thickness in the mid-sagittal plane. The measurement encompassed the thickest echogenic area from one stratum basalis endometrial interface to the other [35]. Additionally, the pulsatility index (PI) and resistive index (RI) of the uterine arteries at the cervical-corporeal level of the uterus were recorded [36]. If the EM pattern was trilaminar and the EM thickness was below 7 mm, autologous PRP treatment was offered after obtaining informed consent. In cases where the EM thickness was below 7 mm and the patients declined PRP treatment but still wished to proceed with embryo transfer, we classified the patients into the control group. This study utilized two methods: intrauterine infusion and hysteroscopic injection. The patient’s choice between the two methods was made by the patients after a thorough discussion. EM thickness would be measured again 2 to 3 days after the treatment of PRP. If the EM thickness remained inadequate, embryo transfer was canceled. However, if the EM thickness exceeded 7 mm, progesterone was administrated, which included oral dydrogesterone (Duphaston^®^, Abbott, Hong Kong, China) and vaginal progesterone gel (Crinone^®^, Merck-Serono, Darmstadt, Germany) for 5 days. The duration of HRT before progesterone administration was 11 to 16 days. Only euploid embryos were transferred under trans-abdominal sonography guidance.

### 2.6. Autologous Plate-Rich Plasma (PRP) Preparation

From each patient, 20 mL of whole blood was drawn using a sterile syringe without anticoagulant and divided into two Acti-PRP tubes (Aeon Biotherapeutics Corp., Taipei, Taiwan). The tubes were then centrifuged at 3600 rpm for 6 min at room temperature (A500, Aeon Biotherapeutics Corp., Taipei, Taiwan). Most of the yellow plasma was removed, leaving approximately 0.5 mL plasma in each tube. The tubes were then inverted at least 30 times to mix the plasma with the buffy coat. After the PRP concentrate solidified, a needle was used to gently stir the concentrate, transforming it into a gelatinous state. This process was repeated three or four times until the PRP concentrate was no longer clotted. Finally, the PRP concentrate was collected using a sterile syringe for infusion or injection into the uterine cavity within 30 min. All procedures were performed following the instructions provided by Aeon Biotherapeutics Corp.

### 2.7. Intrauterine Infusion of Autologous PRP

A 2 mL amount of autologous PRP was infused into the uterine cavity twice, with a 48 h interval between each infusion which was similar to that in our previous study [18]. The autologous PRP was infused directly into the uterine cavity using an embryo transfer catheter (Guardia^TM^ Access^®^, Cook; Bloomington, IN, USA).

### 2.8. Hysteroscopic Injection of Autologous PRP

These patients received intravenous anesthesia. With the guidance of hysteroscopy, 2 mL of autologous PRP was injected into the endometrium at a depth of 2–3 mm in four directions including upper, lower, right, and left side of uterine cavity, 0.5 mL in each direction. The injection was performed using a 19 GA single-lumen ovum aspiration needle (Cook; USA), with its beveled edge serving as a guide.

### 2.9. Outcomes Measurement

The calculation of implantation rate was determined by the number of visible gestational sacs observed through ultrasound divided by the number of embryos transferred. The clinical pregnancy rate was the number of patients with gestational sac(s) with fetal heartbeat(s) on ultrasound scan at 6 or 7 weeks of gestation divided by the number of patients who underwent transfer. The live birth rate was determined by the number of live births after 24 weeks’ gestation divided by the number of patients receiving ET.

### 2.10. Data Analysis

The data analysis was conducted using the software SPSS (v 20.0; IBM Corporation, Armonk, NY, USA). A level of *p* < 0.05 was considered statistically significant for all analyses. The implantation rate was calculated as the number of gestational sacs divided by the number of embryos transferred. Clinical pregnancy was determined by the presence of intrauterine fetal heartbeats observed in transvaginal sonography at 6 or 7 weeks’ gestation [37]. Miscarriage was defined as pregnancy loss occurring before 24 weeks’ gestation. Categorical variables are presented by frequency and percentage, while continuous variables are presented by the median and interquartile range (25th–75th percentile) or mean ± standard deviation. The Kruskal–Wallis test (for continuous variables with non-normal distribution), ANOVA test (for continuous variables with normal distribution), and the chi-squared test (for categorical items) were applied to evaluate the differences among the four studied groups. Bonferroni correction adjusted the *p* value for post hoc multiple comparisons between the four studied groups [significant *p* value will be corrected as 0.0083 (0.05/6)].

## 3. Results

A total of 116 infertile women with thin EM on cycle day 11 to 13 of the menstrual cycle, after undergoing standard HRT for FET, and having experienced at least one previous unsuccessful EFET cycle, including cases where the cycle was canceled due to persistent thin EM or failure to achieve pregnancy after receiving euploid embryo transfer despite thin EM, were enrolled in this study. After explanation, the patients decided which treatment option to receive (intrauterine infusion of PRP, hysteroscopic injection of PRP, or HRT treatment only). Among them, 55 women received an intrauterine infusion of autologous PRP, 38 women received a hysteroscopic injection of autologous PRP, and 23 women chose to only take standard HRT treatment without receiving PRP treatment from the control group. Only euploid embryos were transferred in these EFET cycles. In addition, 30 patients receiving standard HRT for EFET with EM thickness ≥ 7 mm were included in the regular group. The demographic characteristics of the patients are presented in Table 1, showing no significant differences in age, partner’s age, BMI, infertility duration, and number of previous failed IVF cycles among the four groups. Most patients had a history of infertility due to mixed factors, including both male and female factors. Comparing the number of prior uterine surgeries, including D&C, transcervical resection, and myomectomy, it was found that the surgical frequency in the hysteroscopic injection group was significantly higher than that in the regular group.

Table 2 represents the outcomes of PRP treatment. The EM thickness on cycle days 11 to 13 was significantly greater in the regular group compared to the other three groups (9.6 ± 1 mm., 6.0 ± 0.6 mm, 6.1 ± 1.0 mm, and 6.0 ± 0.6 mm, respectively; *p* < 0.001). After receiving intrauterine infusion and hysteroscopic injection of PRP, the EM thickness was 8.2 ± 1.8 mm and 7.5 ± 1.8 mm, respectively (*p* = 0.026). There was a significant increase (*p* < 0.001) in EM thickness compared to before the treatment in both groups. After receiving an intrauterine infusion of PRP, 78.2% of patients (43/55) exhibited an endometrial thickness exceeding 7 mm and subsequently underwent embryo transfer; the EM thickness on ET day was 8.8 ± 1.4 mm. In the group receiving hysteroscopic injection of PRP, 55.3% of patients (21/38) experienced an increase in EM thickness beyond 7 mm, followed by embryo transfer; the EM thickness on ET day was 8.7 ± 1.7 mm. On ET day, patients who received intrauterine infusion of PRP or hysteroscopic injection of PRP both exhibited a significantly thicker EM thickness compared to the control group (*p* < 0.001). The control group only received standard HRT, without using PRP, and the average EM thickness on ET day was 6.7 ± 0.4 mm. In addition, all 23 patients chose to undergo embryo transfer.

The pregnancy outcomes are presented in Table 3. The implantation rate (IR) after EFET was significantly higher in the hysteroscopic injection group compared to the control group (52% vs. 18%, respectively; *p* < 0.001). The clinical pregnancy rate (CPR) in the hysteroscopic injection group did not show statistical differences compared to the control group (52% vs. 22%, respectively), but it exhibited a higher trend. The IR and CPR were also higher in the intrauterine infusion group compared to the control group, although without statistical significance (27% and 33% vs. 18% and 22%, respectively; *p* > 0.05). The live birth rate was significantly higher in the hysteroscopic injection group compared to the control group (38% and 4%, respectively; *p* < 0.01). Among these four groups, the IR, CPR, and live birth rates were the highest in the regular group.

## 4. Discussion

Previous studies have reported that 1.5% to 9.1% of infertile women undergoing ART had an EM thickness of less than 7 mm in the FET cycle and less than 8 mm in the fresh IVF cycle [3,7]. Such thin EM is associated with negative impacts on pregnancy outcomes following embryo transfer. In our study, the implantation rate, clinical pregnancy rate, and live birth rate were significantly higher in the regular group (EM thickness ≥ 7 mm on cycle day 11 to 13) compared to the control group (EM thickness < 7 mm, under conservative HRT) (75%, 70% and 81% vs. 18%, 22%, and 20%, respectively; *p* < 0.001), as shown in Table 3. These findings underscore the negative impact of a thin EM on pregnancy outcomes.

There have been various adjuvants investigated to increase EM thickness, including aspirin, pentoxifylline, sildenafil, vitamin E, L-arginine, extended estradiol usage, human chorionic gonadotropin (hCG), gonadotropin-releasing hormone agonist (GnRHa), electroacupuncture, intrauterine infusion of granulocyte colony-stimulating factor (G-CSF), stem cells and PRP. However, there is currently insufficient evidence to recommend any of these adjuvants mentioned above [6,12,23,28,38].

Based on our previous research on patients with thin EM and repeated implantation failure, intrauterine infusion of PRP could increase the EMT to over 7 mm in 86% of patients [18]. In our current study, intrauterine infusion of autologous PRP significantly increased EM thickness and live birth rate, showing an increase in IR and CPR, although the differences were not statistically significant, as shown in Table 2 and Table 3. Our results are consistent with previous research; however, in the previous studies, embryos transferred included both third-day and fifth-day embryos [12,23], making it difficult to rule out the potential for pregnancy failure due to chromosomal abnormalities in the embryos. In our study, only euploid embryos were transferred, eliminating the interference of embryo aneuploidy.

The hysteroscopic injections were performed on menstrual cycle days 11 to 13 in this study, and the EM thickness increased in 55.3% of patients after the PRP injection, which made it eligible for embryo transfer in the same cycle. The previous method involved hysteroscopic injecting of PRP in the preceding menstrual cycle, followed by embryo transfer in the next cycle [12]. In that study, 75% of the patients had an EM thickness of 7 mm or greater in the subsequent HRT cycle, resulting in a pregnancy rate of 50% after FET. The endometrium undergoes cyclic regeneration and shedding in each menstrual cycle. The cyclic growth of the endometrium is facilitated by endometrial progenitor cells and bone marrow-derived stem cells (BMDSCs) present in the basalis layer of the endometrium [39,40,41]. In a prospective experimental, non-controlled study published in 2016, CD133+ BMDSCs were delivered into the spiral arterioles by catheterization in patients diagnosed with refractory Asherman’s syndrome and/or endometrial atrophy, demonstrating an increase in EM thickness and improvement in neoangiogenesis [42]. The injected CD133+ BMDSCs were detected in the endometrium, confirming the engraftment of stem cells around the endometrial vessels and the proliferation of surrounding cells [43]. A pilot randomized study published in 2023 demonstrated the efficacy of hysteroscopic injection of autologous PRP for patients with refractory thin EM, and the curative effects of PRP may be related to the growth factors in PRP, stimulating the EM stem cells [9,44]. In the current study, 38 patients received the hysteroscopic injection and 21 patients (55.3%) exhibited an EM thickness exceeding 7 mm (with an average of 8.7 mm). Unlike previous studies, in this research, the injection of PRP and embryo transfer occurred within the same cycle, reducing the duration of treatment, and minimizing the time to pregnancy.

To compare intrauterine infusion of autologous PRP with hysteroscopic injection, the advantage of hysteroscopic injection is the ability to deliver a larger volume of PRP (2 mL in our study, up to 40 mL in the literature) compared to the 1–2 mL used in most intrauterine infusion studies [9]. This larger volume increases the number of functional cytokines and growth factors reaching the endometrium. Additionally, hysteroscopic injection allows for the direct delivery of autologous PRP into the subendometrial region, near the endometrial progenitor cells and mesenchymal stem cells (MSCs), under real-time hysteroscopic guidance, without any leakage. In our current study, after receiving an intrauterine infusion of PRP, the EM thickness was greater than in those who received hysteroscopic injection of PRP (8.2 ± 1.8 mm and 7.5 ± 1.8 mm, respectively; *p* = 0.026). Additionally, following intrauterine infusion of PRP, 78.2% of patients had an EM thickness exceeding 7 mm. However, in the hysteroscopic injection of the PRP group, 55.3% of patients had an EM thickness exceeding 7 mm, and these results showed statistical significance (*p*= 0.024). According to the findings of this study, intrauterine infusion was more effective in increasing EM thickness compared to hysteroscopic injection of PRP. On ET day, the EM thickness was significantly higher in the intrauterine infusion and hysteroscopic injection groups compared to the control group (8.8 ± 1.4 mm and 8.7 ± 1.7 mm vs. 6.7 ± 0.4 mm respectively; *p* < 0.001), showing both methods could increase EM thickness. However, after embryo transferring, the hysteroscopic injection group exhibited a significantly higher IR and live birth rate, and a higher trend of CPR compared to the control group. This may be attributed to the direct injection of growth factors contained in PRP near the progenitor cells and stem cells in the basalis layer of endometrium through hysteroscopic injection of PRP, thereby promoting cell growth and facilitating embryo implantation, improving pregnancy outcomes. Further research is needed to confirm this phenomenon and explore the potential reasons.

One limitation of our study is the lack of randomization. The patients themselves decided to receive intrauterine infusion or hysteroscopic injection after a careful and detailed explanation. Factors such as cost, the necessity of anesthesia, and the surgical procedure may have influenced their preference. The similarity in basic characteristics among our patients in the four groups reduces the selection bias, as shown in Table 1. However, this study is not a double-blind study, which could potentially introduce the result biases. Additionally, we still cannot rule out the possibility of selection bias due to the inability to conduct random sampling. This study is a preliminary investigation. Therefore, there was no pre-calculation of the number of enrolled patients, and no power analysis was conducted. If a randomized controlled trial (RCT) is conducted in the future, more precise design considerations will be made regarding this aspect. Other limitations include the variability in the methods used to prepare PRP in the literature, which may affect the comparability of PRP therapy efficacy. In our study, we used one-step centrifugation with mechanical activation of PRP, but different preparation and activation procedures were employed in other studies [10]. Additionally, we did not analyze the platelet concentration or the content of cytokines and growth factors in the PRP. The smaller amount of PRP used for hysteroscopic injection in our study compared to the previous published study [9] may also affect the effectiveness of PRP therapy.

## 5. Conclusions

To the best of our knowledge, this is the first study to compare the efficacy of intrauterine infusion and hysteroscopic injection of autologous PRP for patients with persistent thin EM in euploid FET cycles. According to our results, both methods could significantly increase the EM thickness. Intrauterine infusion of PRP is more effective in increasing endometrial thickness. Compared to the control group, both methods could increase the implantation rate and clinical pregnancy rate. However, only hysteroscopic injection of PRP significantly increases the implantation rate and live birth rate. Nevertheless, until more studies are published confirming the ability of PRP to improve pregnancy outcomes in infertility patients, the use of PRP remains in the research phase and should not be routinely recommended for patients. Future research, including randomized controlled trials (RCTs), should be conducted to investigate the benefits of PRP in infertile patients.

## Figures and Tables

**Table 1:** Patient characteristics.

	Regular Group (EM ≥ 7 mm)	Control Group (EM < 7 mm)	Intrauterine Infusion of PRP	Hysteroscopic Injection of PRP	*p* Value
Patients numbers	30	23	55	38	
Age (years)	38 ± 4.1	39.0 ± 6.6	37.9 ± 6.9	40.0 ± 5.9	0.347
Partner’s age (years)	41.8 ± 6.6	42.7± 7.2	39.0 ± 7.0	41.7 ± 7.3	0.103
BMI (kg/m^2^)	22.1 ± 3.3	21.8 ± 3.0	21.7 ± 2.3	22.4 ± 3.1	0.786
Prior uterine surgery (n)	0.6 ± 0.8 (0–2) ^a^	1.7 ± 1.8 (0–7)	1.5 ± 1.6 (0–5)	1.9 ± 1.6 (0–7) ^a^	0.004
Infertility duration (years)	2.4 (1.0–4.4)	1.6 (1.0–4.6)	3 (1.2–5.1)	3 (2–4.15)	0.322
Number of previous failed IVF cycle (n)	3 (2–5)	3 (2–7)	5 (2–7)	5 (3–8)	0.031
Etiology of infertility (n)					
Male factor	3 (10%)	4 (17%)	4 (7%)	2 (5%)	
Female factor					
Ovarian factor	2 (7%)	8 (35%)	17 (31%)	18 (47%)	
Tubal factor	0	1 (5%)	3 (5%)	0	
Male and female factors	25 (83%)	10 (43%)	31 (57%)	18 (48%)	

Abbreviation: BMI: body mass index; PRP: platelet-rich plasma. Note: Age and BMI are presented as mean ± SD. Infertility duration and number of failed IVF times are presented as median (interquartile range). *p* value through AVNOVA test, Kruskal–Wallis test, or chi-square test as appropriate. ^a^ *p* < 0.01 between the number of prior uterine surgery in regular group and hysteroscopic injection of PRP group.

**Table 2:** The outcomes of PRP treatment.

	Regular Group (EM ≥ 7 mm)	Control Group (EM < 7 mm)	Intrauterine Infusion of PRP	Hysteroscopic Injection of PRP	*p* Value
Patients numbers	30	23	55	38	
EM thickness on cycle day 11 to 13 (mm)	9.6 ± 1.5 ^a,b,c^ (7–12.1)	6.0 ± 0.6 ^a^ (4–6.9)	6.1 ± 1.0 ^b^ (3–6.9)	6.0 ± 0.6 ^c^ (4–6.7)	<0.001
Pulsatility index on cycle day 11 to 13	2.2 ± 0.7	2.1 ± 0.6	2.0 ± 0.5	2.1 ± 0.7	0.805
Resistive index on cycle day 11 to 13	0.8 ± 0.07	0.8 ± 0.07	0.8 ± 0.1	0.8 ± 0.1	0.948
EM thickness after PRP treatment (mm)			8.2 ± 1.8 (4.9–15)	7.5 ± 1.8 (5.1–13)	0.026
Numbers of patients with EM thickness ≥ 7 mm after PRP treatment			43 (78.2%)	21 (55.3%)	0.024
EM thickness on ET day (mm)	11.7 ± 2.2 ^d,e,f^ (8–18.7)	6.7 ± 0.4 ^d,g,h^ (5.3–6.9)	8.8 ± 1.4 ^e,g^ (7–15)	8.7 ± 1.7 ^f,h^ (7–13)	<0.001

Abbreviation: EM: endometrium; ET: embryo transfer; PRP: platelet-rich plasma. *p* value through AVNOVA test or Kruskal–Wallis test as appropriate. Bonferroni correction adjusted the *p* value for post hoc multiple comparisons between the four studied groups [significant *p* value will be corrected as 0.0083 (0.05/6)]. ^a^ *p* < 0.001 between the EM thickness on cycle D11 to 13 in regular group and control group. ^b^ *p* < 0.001 between the EM thickness on cycle D11 to 13 in regular group and intrauterine infusion of PRP group. ^c^ *p* < 0.001 between the EM thickness on cycle D11 to 13 in regular group and hysteroscopic injection of PRP group. ^d^ *p* < 0.001 between the EM thickness on ET day in regular group and control group. ^e^ *p* < 0.001 between the EM thickness on ET day in regular group and intrauterine infusion of PRP group. ^f^ *p* < 0.001 between the EM thickness on ET day in regular group and hysteroscopic injection of PRP group. ^g^ *p* < 0.001 between the EM thickness on ET day in control group and intrauterine infusion of PRP group. ^h^ *p* < 0.001 between the EM thickness on ET day in control group and hysteroscopic injection of PRP group.

**Table 3:** Pregnancy outcomes.

	Regular Group (EM ≥ 7 mm)	Control Group (EM < 7 mm)	Intrauterine Infusion of PRP	Hysteroscopic Injection of PRP	*p* Value
Numbers of patients receiving ET	30	23	43	21	
Total number of embryos transferred	40	40	60	33	
Average number of embryos transferred per patients	1.3 ± 0.5 (1–2)	1.7 ± 0.7 (1–2)	1.4 ± 0.5 (1–2)	1.6 ± 0.5 (1–2)	0.055
Implantation rate (%)	75% (30/40) ^a,b^	18% (7/40) ^a,c^	27% (16/60) ^b^	52% (17/33) ^c^	0.011
Clinical pregnancy rate (%)	70% (21/30) ^d,e^	22% (5/23) ^d^	33% (14/43) ^e^	52% (11/21)	0.001
Live birth rate (%)	67% (20/30) ^f,g^	4% (1/23) ^f,h^	23% (10/43) ^g^	38% (8/21) ^h^	<0.001

Abbreviation: ET: embryo transfer; PRP: platelet-rich plasma. *p* value through Kruskal–Wallis test or Pearson chi-square test as appropriate. Bonferroni correction adjusted the *p* value for post hoc multiple comparisons between the four studied groups [significant *p* value will be corrected as 0.0083 (0.05/6)]. ^a^ *p* < 0.001 between the implantation rate in regular group and control group. ^b^ *p* < 0.001 between the implantation rate in regular group and intrauterine infusion of PRP group. ^c^ *p* < 0.001 between the implantation rate in control group and hysteroscopic injection of PRP group. ^d^ *p* < 0.001 between the clinical pregnancy rate in regular group and control group. ^e^ *p* < 0.001 between the clinical pregnancy rate in regular group and intrauterine infusion of PRP group. ^f^ *p* < 0.001 between the live birth rate in regular group and control group. ^g^ *p* = 0.003 between the live birth rate in regular group and intrauterine infusion of PRP group. ^h^ *p* = 0.0082 between the live birth rate in control group and hysteroscopic injection of PRP group.

## Data Availability

The data presented in this study are available on request from the corresponding author.

## List of Abbreviations

ART	assisted reproductive technology
BMDSCs	bone marrow-derived stem cells
BMI	body mass index
COH	controlled ovarian hyperstimulation
CPR	clinical pregnancy rate
CTGF	connective tissue growth factor
EGT	endothelial growth factor
EFET	euploid frozen embryo transfer
EM	endometrium
ET	embryo transfer
FET	frozen embryo transfer
GnRHa	gonadotropin-releasing hormone agonist
hCG	human chorionic gonadotropin
HGF	hepatocyte growth factor
hr-NGS	high resolution next generation sequencing
HRT	hormone replacement therapy
ICSI	intracytoplasmic sperm injection
IFN	interferon
IGF	insulin-like growth factor
IL	interleukin
IR	implantation rate
MSC	mesenchymal stem cell
PDGF	platelet-derived growth factor
PGT-A	preimplantation genetic testing for aneuploidy
PI	pulsatility index
PRP	platelet-rich plasma
RCT	randomized controlled trial
RI	resistive index
TGF	transforming growth factor
TNF	tumor necrosis factor
VEGF	vascular endothelial growth factor
